# Editorial: The Contrast Sensitivity Function: From Laboratory to Clinic

**DOI:** 10.3389/fnins.2021.783674

**Published:** 2021-10-25

**Authors:** Fang Hou, Zhong-Lin Lu, Peter Bex, Alexandre Reynaud

**Affiliations:** ^1^School of Ophthalmology & Optometry and Eye Hospital, Wenzhou Medical University, Wenzhou, China; ^2^Division of Arts and Sciences, NYU Shanghai, Shanghai, China; ^3^Department of Psychology, Center for Neural Science, New York University, New York, NY, United States; ^4^NYU-ECNU Institute of Brain and Cognitive Sciences at NYU Shanghai, Shanghai, China; ^5^Department of Psychology, Northeastern University, Boston, MA, United States; ^6^McGill Vision Research, McGill University, Montréal, QC, Canada

**Keywords:** contrast sensitivity function, functional vision, spatial frequency, temporal frequency, sensitivity

The contrast sensitivity function (CSF) describes how sensitivity (1/contrast threshold) to narrow-band stimuli varies with spatial and/or temporal frequency. It provides a fundamental characterization of spatial or temporal vision, and reflects the combined effects of limiting factors from the optics of the eye all the way to the response properties of cortical neurons. As the boundary between the visible and invisible narrow-band visual stimuli, the CSF correlates better with real-world daily visual functions than visual acuity (VA). This Research Topic, consisting of a collection of 13 fundamental and clinical oriented research articles, extends our knowledge related to the CSF.

How stimulus contrast at different spatial or temporal frequencies affects the response properties of the visual system is the core of the CSF. Camillo et al. found that the contrast and temporal frequency tunings of mouse V1 neurons are inseparable. The divisive contrast-gain normalization model provided a good fit to their data at high temporal frequencies, but not at low temporal frequencies. The results suggest that different normalization mechanisms exist in the mouse visual cortex and may underlie the different relationships between temporal frequency and spatial contrast tunings at different temporal frequencies.

Using psychophysics and functional MRI, Negishi and Shinomori found that weak color (low saturation) had a stronger inhibitory effect on contrast sensitivity than strong color (high saturation), and the interaction between chromatic and achromatic signals happened in V1. Contrast processing can also be improved by perceptual learning. Xi et al. measured ERP contrast response functions before and after 10 days of perceptual learning. They found that training significantly improved VA and contrast sensitivity, and led to changes at different stages of visual processing.

The CSF is an important functional vision assessment in the clinic. A series of studies on various clinical populations further demonstrated its importance. Rosenkranz et al. validated the performance of qCSF, a Bayesian adaptive method for efficient CSF measurement (Lesmes et al., 2010), in patients with multiple sclerosis (MS). The authors suggest that the qCSF method can be used as a tool to evaluate contrast vision for MS patients. Hoffmann et al. measured visual functions and morphologic parameters of the retina in patients with age-related macular degeneration (AMD). They found that among all the measures of visual function, the CSF correlated best with anatomic features of the retina. Li et al. evaluated the effects of different myopia control lenses on the CSF and VA in a group of myopic children. They found that the lenses with concentrically arranged aspherical lenslets had the least influence on the CSF and VA. Min et al. evaluated effects of Eyetronix Flicker Glass (EFG) treatment on children with amblyopia on the CSF and VA, and concluded that the EFG can be an additional choice for amblyopia therapy. Cheng et al. showed that 0.01% atropine had no significantly detrimental effect on the CSF in myopic adults, suggesting that 0.01% atropine might be safely used for myopia control. Finally, Stalin et al. assessed visual functions and skiing performance in elite skiers with visual impairments, and found that the CSF did not add any predictive value on skiing performance.

In clinical settings, it is important to measure the CSF quickly, accurately and reliably. Some of the submitted works explored new methods for efficient CSF measurement. The Campbell-Robson chart is widely used to illustrate the CSF as the boundary between the visible and invisible narrow band visual stimuli, and this border might be used to estimate the CSF. However, Tardif et al. examined this putative idea and concluded that the Campbell-Robson chart did not accurately quantify the CSF. Xiong et al. provided a method to simulate visual quality of patients with low vision based on clinical measures of VA and CSF. Their method successfully predicted reading performance and text visibility under a wide range of low vision conditions. Xu et al. developed a Bayesian adaptive method (qVFM) to estimate the contrast sensitivity map over the visual field. They demonstrated that the qVFM method could accurately and efficiently evaluate the contrast sensitivity visual field map in normal observers. Finally, because current CSF tests are too boring to sustain young children's interest, Elfadaly et al. developed a gamified, child-friendly CSF assessment and showed that it successfully assessed CSF in young children.

Altogether this collection of articles emphasizes the importance of measuring the CSF to assess visual function in both basic research and clinical settings. It presents some methods to perform and improve those measures, and considers their interpretation and implications.

## Author Contributions

All authors contributed to manuscript writing and revision, read, and approved the submitted version.

## Funding

This work was supported by the National Natural Science Foundation of China (NSFC81600764 to FH) and the Department of Human Resources and Social Security of Zhejiang Province (Qianjiang Talent Project, QJD1803028 to FH).

## Conflict of Interest

PB and Z-LL hold equity shares in Adaptive Sensory Technology, Inc. and have patents related to the qCSF technology. The remaining authors declare that the research was conducted in the absence of any commercial or financial relationships that could be construed as a potential conflict of interest.

## Publisher's Note

All claims expressed in this article are solely those of the authors and do not necessarily represent those of their affiliated organizations, or those of the publisher, the editors and the reviewers. Any product that may be evaluated in this article, or claim that may be made by its manufacturer, is not guaranteed or endorsed by the publisher.
